# Effects of Exercise-Based Interventions on Self-Reported Sleep Outcomes in Parkinson’s Disease: A Systematic Review and Meta-Analysis of Randomized Controlled Trials

**DOI:** 10.3390/bioengineering13091027

**Published:** 2026-09-03

**Authors:** Chengshuo Zhang, Xinyuan Zhang, Lei Cao, Jiaxin Li, Zhengqiu Zhang, Wanli Zang

**Affiliations:** 1 School of Physical Education, Henan University of Science and Technology, Luoyang 471023, China; 2 School of Physical Education, Soochow University, Suzhou 215021, China

**Keywords:** exercise-based intervention, self-reported sleep outcomes, Parkinson’s disease, randomized controlled trial, systematic review, meta-analysis

## Abstract

Objective: To evaluate the effects of exercise-based interventions on self-reported sleep outcomes in patients with Parkinson’s disease (PD) and to assess the certainty of the available evidence. Methods: PubMed, Web of Science, Scopus, the Cochrane Central Register of Controlled Trials (CENTRAL), and China National Knowledge Infrastructure (CNKI) were searched from inception to 17 August 2026. The review protocol was registered with INPLASY (INPLASY202480029). Risk of bias was assessed using the Cochrane Risk of Bias tool (RoB 1). Random-effects meta-analyses were performed using Hedges’ *g*, with between-study variance estimated by restricted maximum likelihood. Exploratory subgroup analyses and univariable meta-regression were used to investigate heterogeneity, and sensitivity analyses were conducted to assess the robustness of the findings. Certainty of evidence was assessed using Grading of Recommendations Assessment, Development and Evaluation (GRADE). Results: Fifteen randomized controlled trials involving 696 participants were included. Exercise-based interventions were associated with improved self-reported sleep outcomes (standardized mean difference [SMD] = −0.75, 95% confidence interval [CI] −1.28 to −0.23; *p* = 0.0084), although between-study heterogeneity was substantial (I^2^ = 82.4%). Instrument-stratified analyses showed no statistically significant differences across sleep questionnaires. Sensitivity analyses restricted to studies using the two most frequently reported sleep questionnaires and leave-one-out analyses yielded estimates consistent in direction with the primary analysis. Subgroup analyses showed no statistically significant differences across exercise intensity, session duration, weekly frequency, intervention period, or exercise type. None of the continuous moderators examined in univariable meta-regression was statistically significant. Conclusions: Exercise-based interventions may improve self-reported sleep outcomes in patients with PD, but the certainty of evidence is very low, and substantial unexplained heterogeneity limits the precision and clinical interpretability of the pooled estimate. Current evidence is insufficient to identify an optimal exercise prescription. Future adequately powered trials should incorporate standardized exercise reporting and objective sleep assessments.

## 1. Introduction

Parkinson’s disease (PD), also known as paralysis agitans, is the second most common and one of the fastest-growing neurodegenerative disorders worldwide, predominantly affecting middle-aged and older adults [1,2]. Its pathological hallmarks include progressive loss of dopaminergic neurons in the substantia nigra pars compacta and the formation of Lewy bodies [1]. Clinically, PD is characterized not only by motor symptoms, including bradykinesia, resting tremor, rigidity, and postural and gait abnormalities, but also by a broad range of non-motor manifestations, such as cognitive impairment, anxiety, depression, sleep disturbances, and gastrointestinal symptoms [3,4]. Among these non-motor manifestations, sleep disturbances are particularly relevant because they may further compromise daytime functioning, quality of life, and long-term disease management. Together with the progressive nature of PD and its associated long-term care needs, these problems contribute substantially to the clinical and societal burden of the disease [5,6,7].

Sleep disturbances in PD are commonly managed using pharmacological approaches, including adjustment of dopaminergic therapy to alleviate nocturnal motor symptoms [8]. However, pharmacological management may provide incomplete relief and may be limited by treatment-related adverse effects, highlighting the need for complementary non-pharmacological strategies. Sleep hygiene education and other non-pharmacological approaches are therefore important components of sleep management in PD [9]. Exercise-based interventions are particularly relevant because exercise has been associated with improvements in several sleep-related outcomes in healthy adults, older adults, and individuals with chronic conditions. Nevertheless, whether these broader exercise–sleep findings can be directly generalized to patients with PD remains uncertain. Sleep disturbances in PD are influenced by disease-specific factors, including nocturnal motor symptoms, tremor, rigidity, dopaminergic medication, autonomic dysfunction, mood symptoms, and neurodegenerative alterations in sleep-regulating circuits [10,11,12].

Although randomized controlled trials (RCTs) have investigated exercise-based interventions for self-reported sleep outcomes in PD, their findings remain inconsistent. Interpretation of the existing evidence is further complicated by substantial clinical and methodological diversity, including differences in participant and disease characteristics, exercise modality and dose, comparator conditions, and sleep assessment instruments. In particular, the self-reported sleep questionnaires used across trials assess related but non-identical aspects of sleep, which may contribute to variability in standardized effect estimates. Consequently, the magnitude and certainty of the overall effect, the extent to which sleep measurement instruments contribute to heterogeneity, and whether exercise characteristics are associated with differences in intervention effects remain unclear.

Accordingly, this systematic review and meta-analysis aimed to quantify the effects of exercise-based interventions on self-reported sleep outcomes in patients with PD, assess the certainty of the available evidence, and explore potential sources of between-study heterogeneity. We hypothesized that exercise-based interventions would be associated with improved self-reported sleep outcomes compared with control conditions.

## 2. Materials and Methods

This systematic review and meta-analysis was conducted and reported in accordance with the Preferred Reporting Items for Systematic Reviews and Meta-Analyses (PRISMA) 2020 statement. The review protocol was prospectively registered with the International Platform of Registered Systematic Review and Meta-analysis Protocols (INPLASY; registration number INPLASY202480029). The review methods were based on the registered protocol, and any post-registration methodological amendments are described in the relevant sections below.

### 2.1. Eligibility Criteria

Studies were eligible if they met the following criteria.

1. Participants: Adults with clinically diagnosed idiopathic PD according to established diagnostic criteria applicable at the time of the original study.

2. Interventions and comparators: The experimental group received a structured exercise-based intervention with sufficient information to characterize the intervention protocol. Eligible comparators included usual care, no-exercise control, wait-list or delayed intervention, supportive care, conventional rehabilitation, health education, or sham/attention-control conditions, provided that the comparator did not include the target exercise intervention being evaluated in the experimental group.

3. Outcomes: Studies were required to report at least one validated self-reported sleep outcome, including measures of sleep quality, insomnia symptoms, or Parkinson’s disease-related nocturnal sleep disturbance [13,14,15,16,17].

4. Study design: RCTs.

Studies were excluded if:

1. The full text was unavailable or sufficient quantitative data for effect-size estimation could not be obtained;

2. They were conference abstracts, case reports, or non-randomized studies; 

3. They were published in languages other than English or Chinese; or

4. They represented duplicate publications or multiple reports of the same study population, in which case the report providing the most complete data relevant to the review was retained.

Full-text dissertations were eligible if they otherwise met all other eligibility criteria and provided sufficient data for quantitative synthesis.

The review was restricted to English- and Chinese-language reports because these were the languages in which the review team could reliably conduct full-text eligibility assessment and data extraction. Relative to the registered protocol, the eligibility criteria for comparator conditions and validated self-reported sleep outcomes were operationally refined during study selection to ensure consistent application of the eligibility criteria. These decisions were finalized before effect-size calculation and quantitative synthesis and were not based on the magnitude or direction of study effects.

### 2.2. Literature Search Strategy

A comprehensive literature search was conducted in PubMed, Web of Science Core, Scopus, the Cochrane Central Register of Controlled Trials (CENTRAL) via the Cochrane Library, and the China National Knowledge Infrastructure (CNKI) from database inception to 17 August 2026. Search strategies were adapted to the indexing systems and syntax of each database. For PubMed and CENTRAL, Medical Subject Headings (MeSH) were combined with free-text terms, whereas database-specific topic or title/abstract/keyword fields were used for Web of Science, Scopus, and CNKI. The search primarily combined terms related to PD and exercise interventions. Trial-design terms were additionally applied in PubMed, Web of Science, Scopus, and CNKI. In CENTRAL, the search was restricted to the Trials database rather than using an additional trial-design search block. To maximize search sensitivity, sleep-related terms were not included as a separate mandatory search concept because eligible sleep outcomes could have been reported as secondary outcomes and might therefore not have been adequately represented in titles, abstracts, or indexing terms. No publication-date restrictions were applied. Complete database-specific search strategies are provided in Supplementary Table S1. In addition, the reference lists of relevant systematic reviews, meta-analyses, and eligible studies were screened to identify additional potentially eligible reports. Relative to the registered protocol, the database set was revised during the review process; the final searches were conducted in the five databases reported above.

### 2.3. Literature Screening and Data Extraction

Two reviewers (C.Z. and J.L.) independently screened the retrieved records according to the eligibility criteria described above. Duplicate records were removed before screening. Titles and abstracts were screened first, followed by full-text assessment of potentially eligible reports. Disagreements were resolved through discussion and, when necessary, adjudicated by a third reviewer (L.C.). When essential information was unclear or unavailable, attempts were made to obtain additional information from the study reports, Supplementary Materials, or study authors.

The same two reviewers (C.Z. and J.L.) independently extracted data using a standardized data-extraction form. Extracted information included study characteristics (e.g., first author, publication year, and country), participant characteristics (e.g., sample size, age, sex, disease duration, and Hoehn and Yahr stage), and intervention and comparator characteristics (e.g., exercise type, exercise intensity, session duration, weekly frequency, intervention period, and control condition). Sleep-outcome data included the assessment instrument, group sample sizes, and numerical data required for quantitative synthesis, such as means and standard deviations at baseline and post-intervention or change-score data when reported. Any discrepancies in data extraction were resolved through discussion and consultation with a third reviewer when necessary.

### 2.4. Risk-of-Bias Assessment

Two independent reviewers (C.Z. and J.L.) assessed the risk of bias in the included RCTs using the Cochrane Risk of Bias tool (RoB 1). The assessment covered seven domains: random sequence generation, allocation concealment, blinding of participants and personnel, blinding of outcome assessment, incomplete outcome data, selective reporting, and other bias. Each domain was judged as having a low, high, or unclear risk of bias based on the information reported in the included studies. Disagreements between the two reviewers were resolved through discussion and, when necessary, adjudicated by a third reviewer (X.Z.).

### 2.5. Statistical Methods

Statistical analyses were performed using R version 4.5.2 (R Foundation for Statistical Computing, Vienna, Austria) with the meta package (version 8.2.1) and the metafor package (version 4.8.0). Because clinical and methodological heterogeneity was anticipated across studies, random-effects models were used for all meta-analyses irrespective of the magnitude of statistical heterogeneity [18]. The included validated sleep instruments were considered to assess related dimensions within the broader construct of self-reported sleep outcomes and were therefore synthesized quantitatively. To account for differences in measurement scales across instruments, standardized mean differences (SMDs), expressed as Hedges’ *g* with 95% confidence intervals (CIs), were used as the effect measure. Analyses were based on between-group differences in change from baseline. For consistency across studies, change scores were calculated from the reported baseline and post-intervention values. The standard deviation (*SD*) of the change score was derived from the baseline and post-intervention SDs using the following formula:

$$SD_{change}=\sqrt{SD_{pre}^{2}+SD_{post}^{2}-2r\left(SD_{pre}\right)\left(SD_{post}\right)}$$

where *r* represents the pre-post correlation coefficient and was set at 0.5. Score directions were harmonized across instruments so that negative effect estimates consistently favored the exercise intervention.

Between-study variance (τ^2^) was estimated using restricted maximum likelihood (REML), and confidence intervals for pooled effects were calculated using the Hartung–Knapp method with an ad hoc variance correction that prevented the Hartung–Knapp standard error from being smaller than the conventional random-effects standard error [19,20]. Statistical heterogeneity was assessed using Cochran’s Q test, I^2^, and τ^2^. Instrument-stratified analyses were performed to examine whether effect estimates differed according to the sleep questionnaire used, and a sensitivity analysis was conducted after restricting the dataset to studies using the two most frequently reported instruments, the Parkinson’s Disease Sleep Scale (PDSS) [21,22,23,24] and Pittsburgh Sleep Quality Index (PSQI) [25,26,27,28,29,30,31,32].

Exploratory subgroup analyses were conducted according to comparator type, exercise intensity, session duration, weekly exercise frequency, intervention period, and exercise type. Comparator conditions were classified as active/structured or inactive/usual-care according to whether the control group received a structured exercise, rehabilitation, behavioral/educational, sham, or attention-control intervention beyond usual care. Exercise intensity was categorized as low, low-to-moderate, moderate, moderate-to-high, or high, without applying a cross-scale numerical conversion of perceived-exertion measures. Session duration was categorized as 20 to <40, 40 to <60, or 60–90 min; weekly exercise frequency as ≤2, 3, or ≥4 sessions/week; and intervention period as <12, 12 to <24, or ≥24 weeks. Exercise type was classified as traditional Chinese exercise, yoga, aerobic exercise, multicomponent exercise, or resistance exercise. Subgroup categories were based on the reported intervention characteristics and their distribution across trials to preserve clinical interpretability while avoiding excessively sparse strata. Subgroups represented by only one study were not quantitatively pooled, and a common estimate of between-study variance was assumed across subgroups. Three separate univariable mixed-effects meta-regression models were fitted for session duration, weekly exercise frequency, and intervention period using REML estimation and ad hoc Knapp–Hartung inference. Given the limited number of studies and the use of study-level characteristics, meta-regression findings were interpreted as exploratory [33].

Robustness of the overall pooled estimate was examined using leave-one-out sensitivity analysis, in which each study was sequentially omitted and the pooled effect and heterogeneity statistics were recalculated. A contour-enhanced funnel plot and Egger’s regression test were used to assess funnel-plot asymmetry and potential small-study effects. All statistical tests were two-sided, with *p* < 0.05 considered statistically significant.

### 2.6. Certainty of Evidence Assessment

The certainty of evidence for the primary outcome was assessed using the Grading of Recommendations Assessment, Development and Evaluation (GRADE) approach. Evidence from RCTs initially started at high certainty and was evaluated across five domains: risk of bias, inconsistency, indirectness, imprecision, and publication bias. Two reviewers independently performed the GRADE assessment, and disagreements were resolved through discussion and, when necessary, consultation with a third reviewer. Certainty was classified as high, moderate, low, or very low. For imprecision, the confidence interval and optimal information size were considered; in the absence of an outcome-specific threshold applicable across the different sleep instruments, the GRADE rule-of-thumb for continuous outcomes was used as a supplementary criterion [34].

## 3. Results

**Figure 1:**
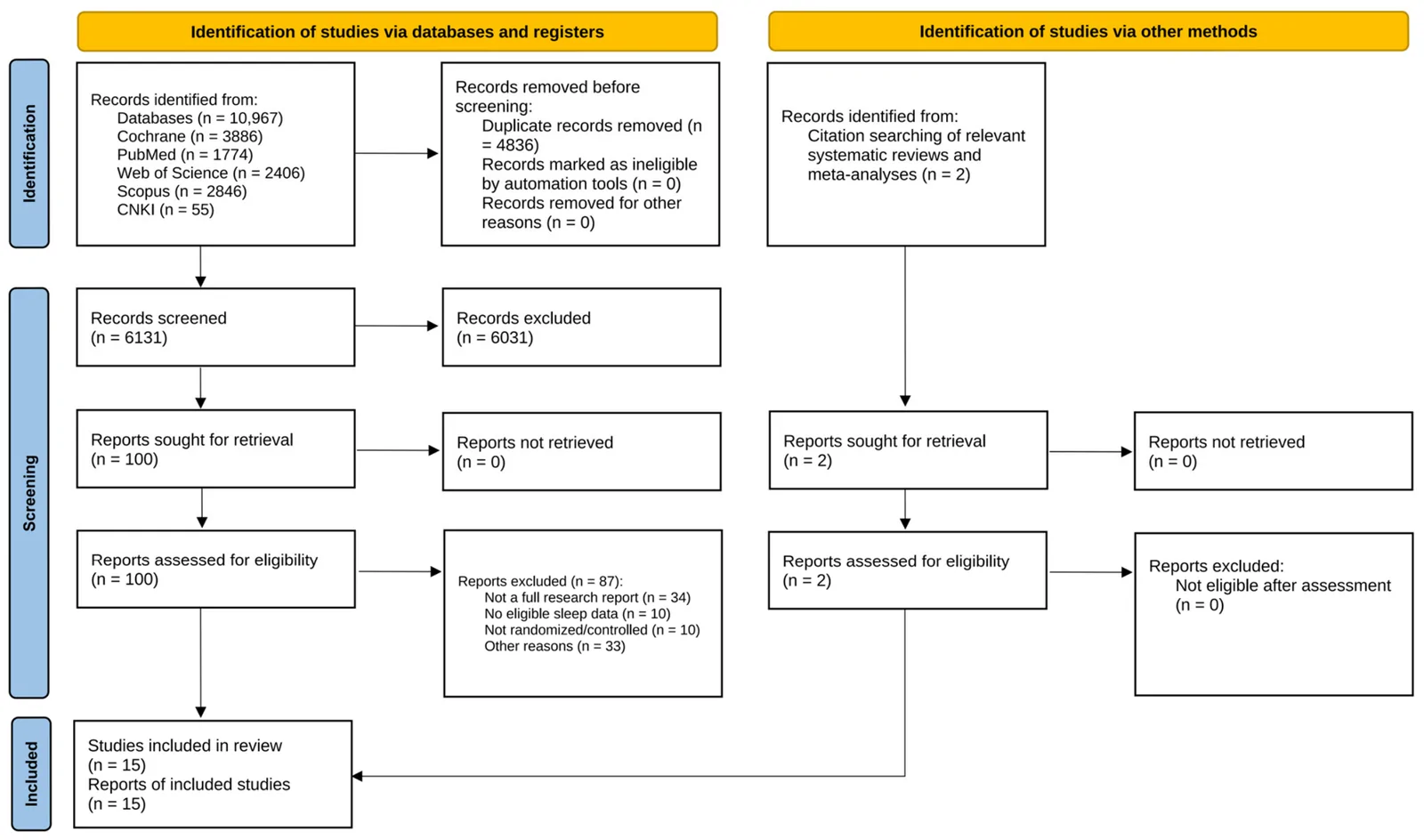
PRISMA 2020 flow diagram of the study selection process.

A total of 10,967 records were identified through database searches. After removal of 4836 duplicate records, 6131 records underwent title and abstract screening, and 100 reports were retrieved for full-text assessment. Of these, 87 were excluded, primarily because they were not eligible full research reports, did not report an eligible self-reported sleep outcome, did not meet the RCT design criterion, or failed to meet other eligibility criteria related to the study population, intervention or comparator, data availability, or duplicate reporting. Thirteen studies identified through database searching met the eligibility criteria. Citation searching of relevant systematic reviews and meta-analyses identified two additional eligible studies. Overall, 15 studies were included in the systematic review and meta-analysis. Detailed reasons for full-text exclusion are presented in Figure 1.

### 3.1. Characteristics of Included Studies

Table 1 summarizes the characteristics of the 15 included RCTs [21,22,23,24,25,26,27,28,29,30,31,32,35,36,37], comprising a total of 696 participants. The studies were conducted across several countries and regions and showed considerable variation in participant characteristics, including age, disease duration, and disease severity, as well as in the type and structure of the exercise interventions. According to the intervention classification used in this review, eight studies evaluated traditional Chinese exercise, two evaluated yoga, two evaluated aerobic exercise, two evaluated multicomponent exercise, and one evaluated resistance exercise, with additional variation in session duration, weekly exercise frequency, and overall intervention period. Four self-reported sleep instruments were used across the included trials: the PSQI in eight studies, the PDSS in four studies, the Parkinson’s Disease Sleep Scale-2 (PDSS-2) in two studies, and the Insomnia Severity Index (ISI) in one study. Detailed participant characteristics, intervention protocols, comparator conditions, and sleep outcome measures are presented in Table 1.

### 3.2. Results of the Risk-of-Bias Assessment

**Figure 2:**
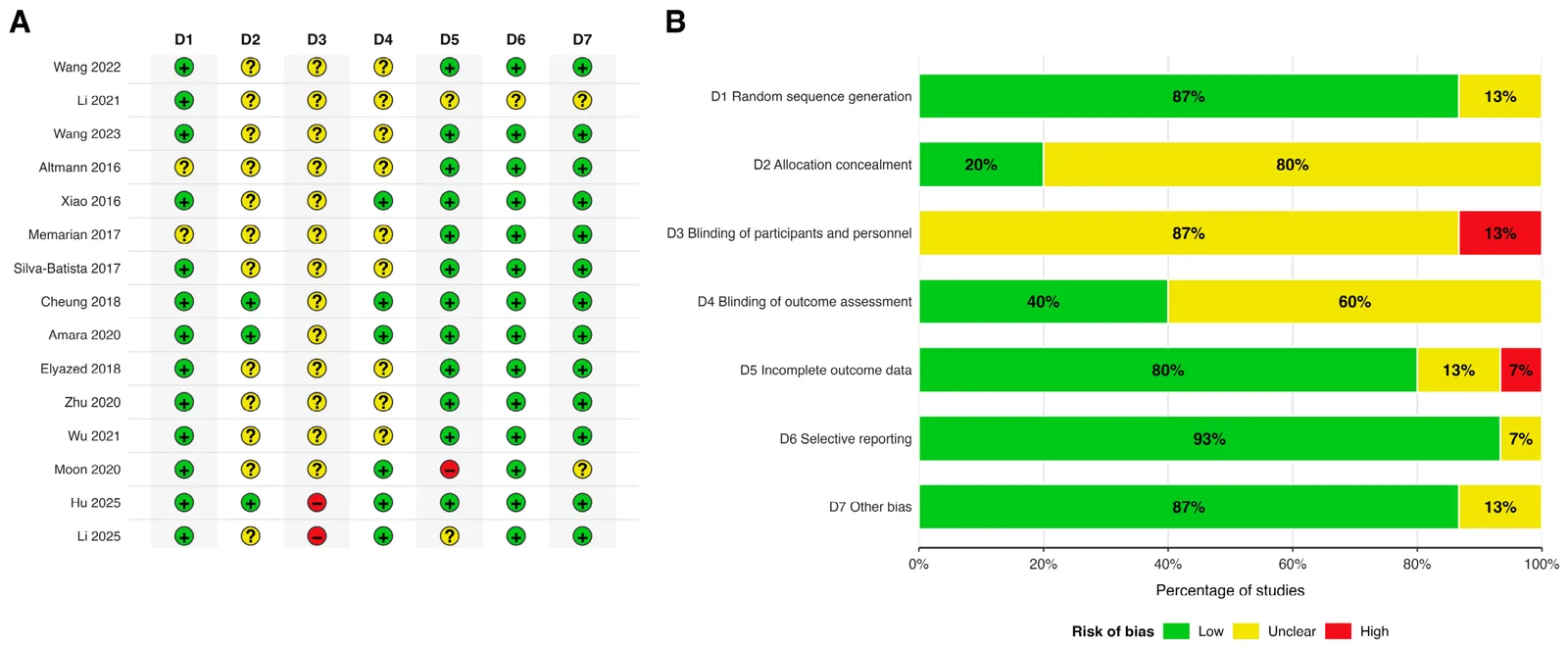
Risk-of-bias assessment of the included studies [21,22,23,24,25,26,27,28,29,30,31,32,35,36,37]. (**A**) Traffic-light plot showing domain-level risk-of-bias judgments for each included study. Green circles containing “+” indicate low risk of bias; yellow circles containing “?” indicate unclear risk of bias; and red circles containing “−” indicate high risk of bias. (**B**) Summary plot showing the percentage distribution of low-, unclear-, and high-risk judgments across the seven RoB 1 domains. D1, random sequence generation; D2, allocation concealment; D3, blinding of participants and personnel; D4, blinding of outcome assessment; D5, incomplete outcome data; D6, selective reporting; D7, other bias.

The risk-of-bias assessment [21,22,23,24,25,26,27,28,29,30,31,32,35,36,37] showed that unclear judgments were concentrated primarily in allocation concealment, blinding of participants and personnel, and blinding of outcome assessment. Specifically, 80% of studies were judged to have an unclear risk of bias for allocation concealment, while 87% and 60% were rated as unclear for blinding of participants and personnel and blinding of outcome assessment, respectively. In addition, 13% of studies were judged to have a high risk of bias for blinding of participants and personnel. In contrast, low-risk judgments predominated for random sequence generation (87%), incomplete outcome data (80%), selective reporting (93%), and other bias (87%). A small proportion of studies were rated as high risk for incomplete outcome data (7%). Overall, unclear or high-risk judgments were concentrated in allocation concealment and blinding-related domains, whereas low-risk judgments predominated in the remaining domains (Figure 2).

### 3.3. Meta-Analysis Results

#### 3.3.1. Overall Effect of Intervention

**Figure 3:**
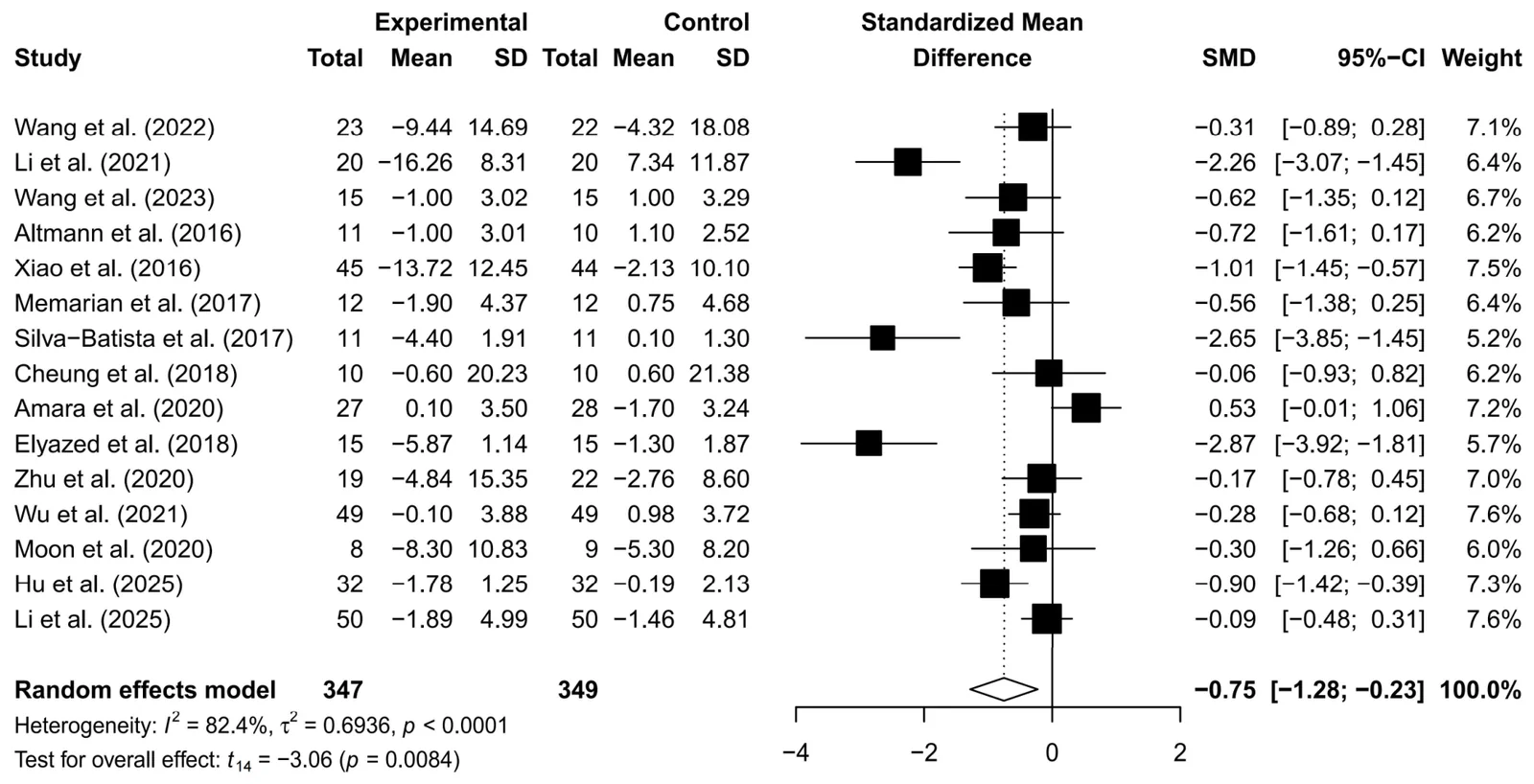
Forest plot of the overall effect of exercise-based interventions on self-reported sleep outcomes in patients with Parkinson’s disease [21,22,23,24,25,26,27,28,29,30,31,32,35,36,37].

After harmonization of score directions, negative SMDs favored exercise-based interventions. The primary random-effects meta-analysis included 15 studies with 696 participants and showed a statistically significant effect of exercise-based interventions on self-reported sleep outcomes in patients with PD (SMD = −0.75, 95% CI −1.28 to −0.23; *p* = 0.0084) [21,22,23,24,25,26,27,28,29,30,31,32,35,36,37]. Substantial between-study heterogeneity was observed (I^2^ = 82.4%; τ^2^ = 0.6936; *p* < 0.0001 for heterogeneity). The overall meta-analysis is presented in Figure 3.

Instrument-stratified analyses were conducted according to the sleep measurement instrument (Supplementary Figure S1). Four studies using the Parkinson’s Disease Sleep Scale (PDSS) [21,22,23,24] yielded a pooled SMD of −0.68 (95% CI −2.31 to 0.96; I^2^ = 85.3%), whereas eight studies using the Pittsburgh Sleep Quality Index (PSQI) [25,26,27,28,29,30,31,32] yielded a pooled SMD of −0.57 (95% CI −1.28 to 0.14; I^2^ = 78.1%). Two studies using the PDSS-2 [36,37] yielded a pooled SMD of −0.70 (95% CI −8.19 to 6.78). The test for subgroup differences was not statistically significant (χ^2^ = 0.06, df = 2, *p* = 0.9707). One study using the ISI [35] represented a single-study instrument category and was not quantitatively pooled as a separate subgroup.

In a sensitivity analysis restricted to the 12 studies using the two most frequently reported sleep measures, the PDSS or PSQI, the pooled effect remained statistically significant (SMD = −0.60, 95% CI −1.15 to −0.06; *p* = 0.0333), with substantial heterogeneity (I^2^ = 79.6%; τ^2^ = 0.5403; *p* < 0.0001 for heterogeneity) (Supplementary Figure S2) [21,22,23,24,25,26,27,28,29,30,31,32]. Within this restricted dataset, the pooled estimates were −0.68 (95% CI −2.31 to 0.96) for PDSS and −0.58 (95% CI −1.30 to 0.14) for PSQI, with no statistically significant difference between the two subgroups (χ^2^ = 0.03, df = 1, *p* = 0.8632).

Leave-one-out sensitivity analyses were performed by sequentially excluding each of the 15 studies (Supplementary Figure S3) [21,22,23,24,25,26,27,28,29,30,31,32,35,36,37]. The recalculated pooled SMDs ranged from −0.85 to −0.61, and all estimates remained negative, with all corresponding 95% CIs excluding zero. Heterogeneity remained substantial across the analyses, with I^2^ values ranging from 78.5% to 83.7% and τ^2^ values ranging from 0.4471 to 0.7717. An exploratory subgroup analysis according to comparator type showed no statistically significant difference between studies using active/structured versus inactive/usual-care comparators (χ^2^ = 0.18, df = 1, *p* = 0.6719), with substantial heterogeneity persisting within both subgroups (Supplementary Figure S4) [21,22,23,24,25,26,27,28,29,30,31,32,35,36,37].

#### 3.3.2. Subgroup Analyses by Exercise Characteristics

##### Exercise Intensity

**Figure 4:**
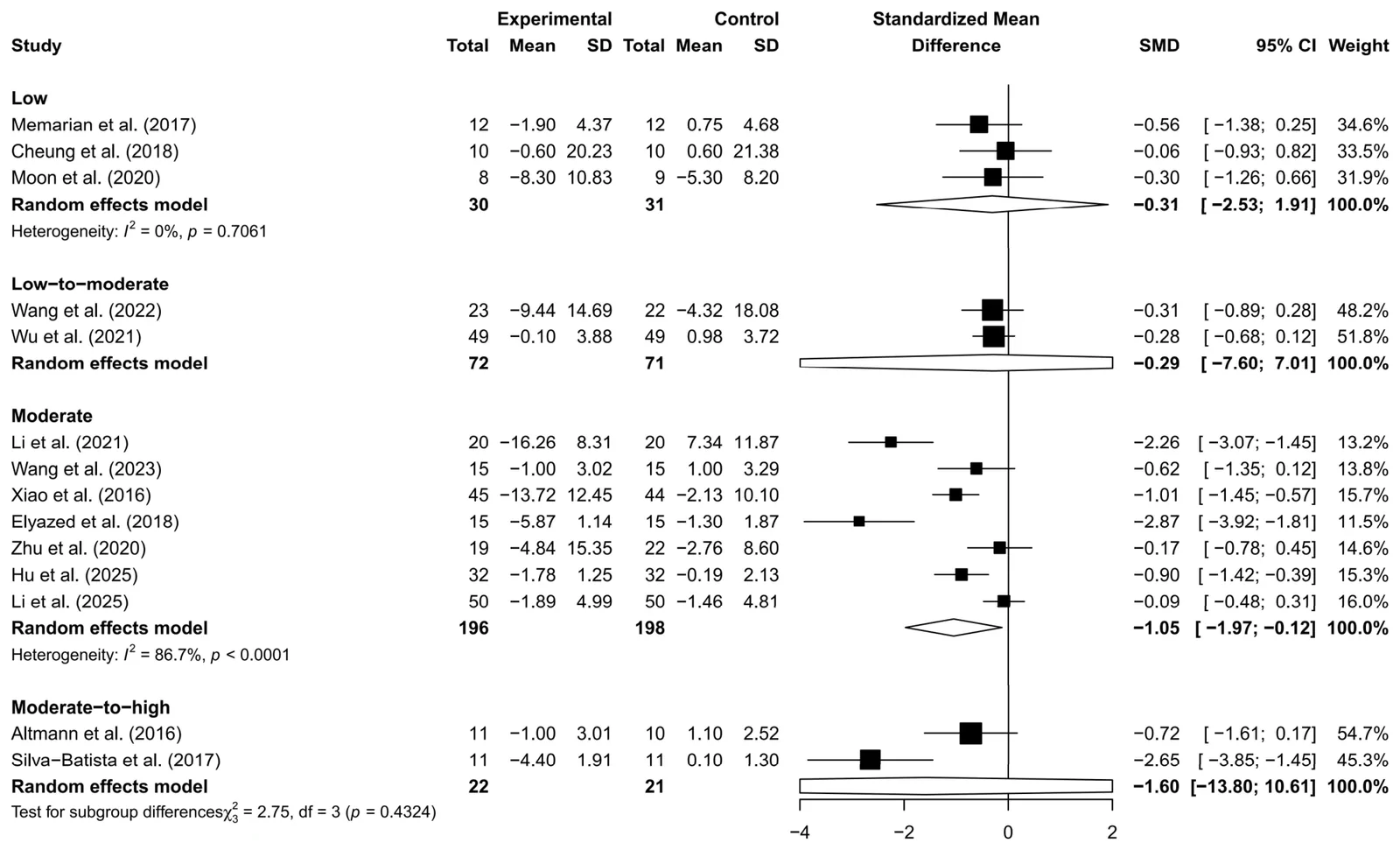
Subgroup Analysis of Exercise Intensity [21,22,23,24,25,27,28,29,30,31,32,35,36,37].

The subgroup analysis according to exercise intensity included 14 studies. Three studies were classified as low intensity [21,30,36], two as low-to-moderate intensity [23,32], seven as moderate intensity [22,24,27,28,29,35,37], and two as moderate-to-high intensity [25,31]. One high-intensity study [26] represented a single-study category and was therefore not quantitatively pooled. For low-intensity exercise, the pooled effect estimate was SMD = −0.31 (95% CI −2.53 to 1.91; I^2^ = 0%). The low-to-moderate-intensity subgroup yielded an SMD of −0.29 (95% CI −7.60 to 7.01). For moderate-intensity exercise, the pooled effect estimate was SMD = −1.05 (95% CI −1.97 to −0.12; I^2^ = 86.7%). The moderate-to-high-intensity subgroup yielded an SMD of −1.60 (95% CI −13.80 to 10.61). The test for subgroup differences was not statistically significant (χ^2^ = 2.75, df = 3, *p* = 0.4324). Detailed results are presented in Figure 4.

##### Session Duration

**Figure 5:**
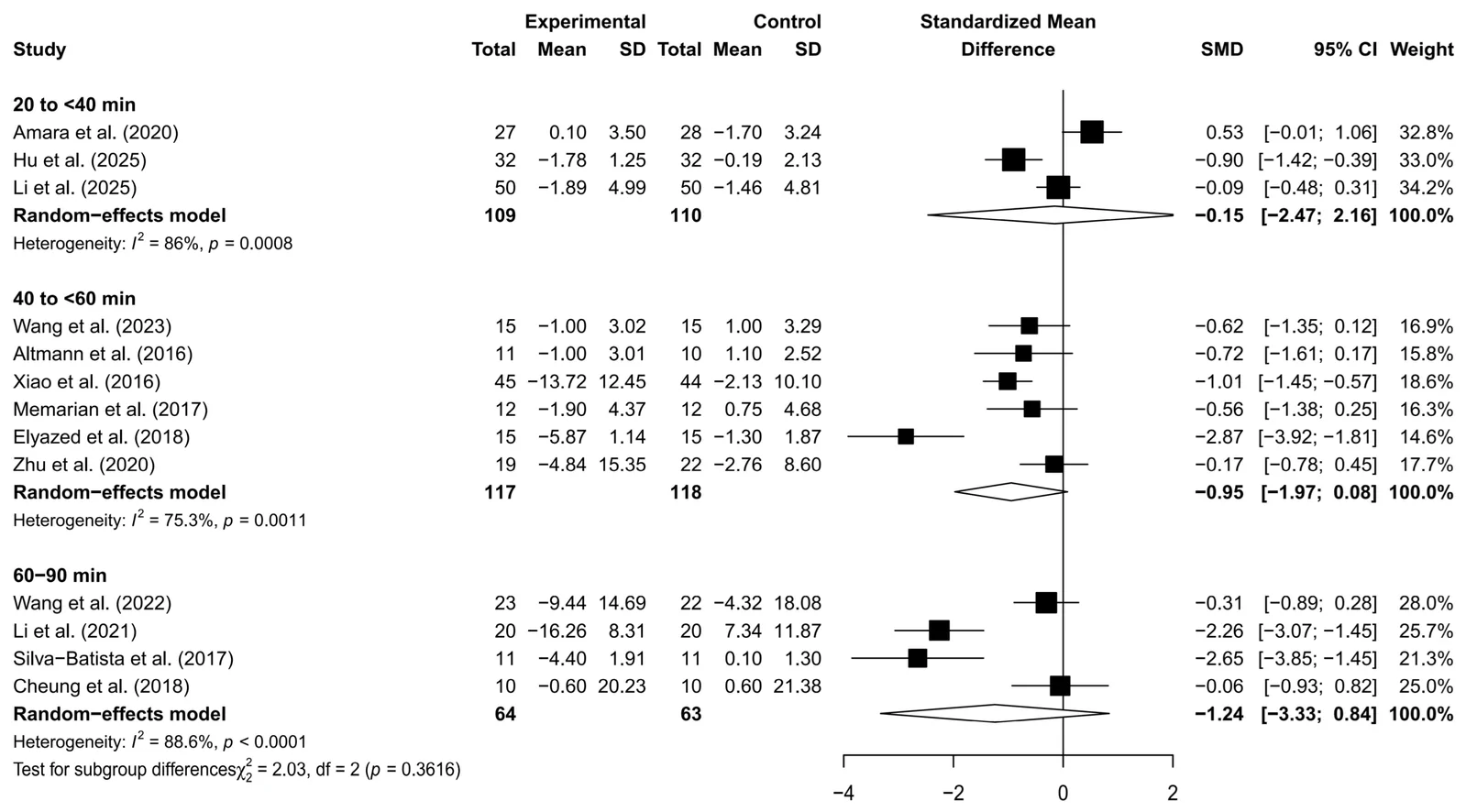
Subgroup Analysis of Exercise Duration per Session [21,22,23,24,25,26,27,28,29,30,31,35,37].

Thirteen studies were included in the subgroup analysis by session duration. Three studies had session durations of 20 to <40 min [26,27,28], six had durations of 40 to <60 min [24,25,29,30,35,37], and four had durations of 60–90 min [21,22,23,31]. Two studies [32,36] for which a single fixed session duration could not be determined were not included in this subgroup analysis. For interventions lasting 20 to <40 min per session, the pooled effect estimate was SMD = −0.15 (95% CI −2.47 to 2.16; I^2^ = 86.0%). For interventions lasting 40 to <60 min, the pooled SMD was −0.95 (95% CI −1.97 to 0.08; I^2^ = 75.3%). For interventions lasting 60–90 min, the pooled effect estimate was SMD = −1.24 (95% CI −3.33 to 0.84; I^2^ = 88.6%). The test for subgroup differences was not statistically significant (χ^2^ = 2.03, df = 2, *p* = 0.3616). Detailed results are presented in Figure 5.

##### Weekly Exercise Frequency

**Figure 6:**
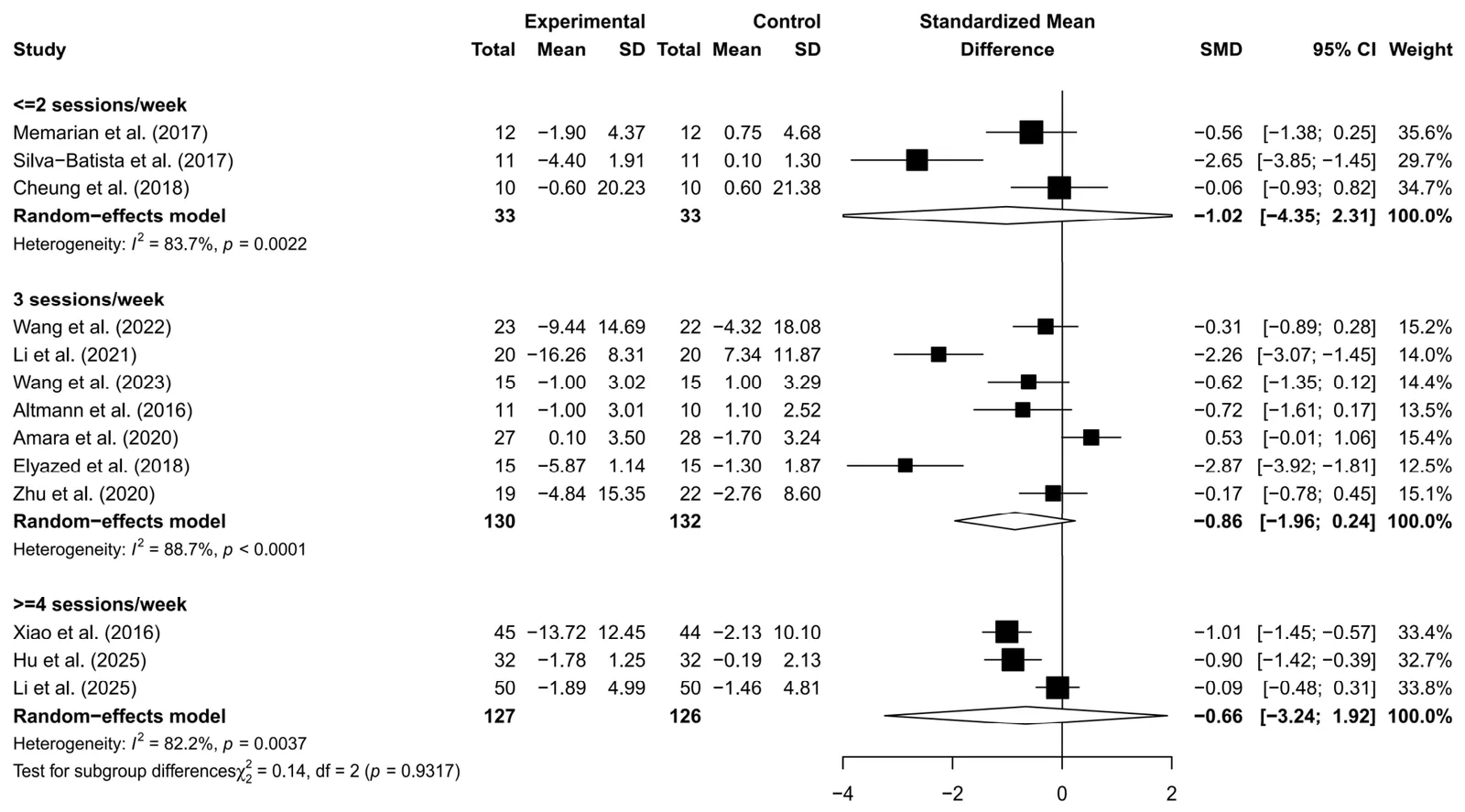
Subgroup Analysis of Intervention Frequency [21,22,23,24,25,26,27,28,29,30,31,35,37].

Thirteen studies were included in the subgroup analysis by weekly exercise frequency. Three studies were classified as ≤2 sessions/week [21,30,31], seven as 3 sessions/week [22,23,24,25,26,29,35], and three as ≥4 sessions/week [27,28,37]. Two studies [32,36] for which a single fixed weekly exercise frequency could not be determined were not included in this subgroup analysis. For interventions performed ≤ 2 sessions/week, the pooled effect estimate was SMD = −1.02 (95% CI −4.35 to 2.31; I^2^ = 83.7%). For interventions performed 3 sessions/week, the pooled SMD was −0.86 (95% CI −1.96 to 0.24; I^2^ = 88.7%). For interventions performed ≥ 4 sessions/week, the pooled effect estimate was SMD = −0.66 (95% CI −3.24 to 1.92; I^2^ = 82.2%). The test for subgroup differences was not statistically significant (χ^2^ = 0.14, df = 2, *p* = 0.9317). Detailed results are presented in Figure 6.

##### Intervention Period

**Figure 7:**
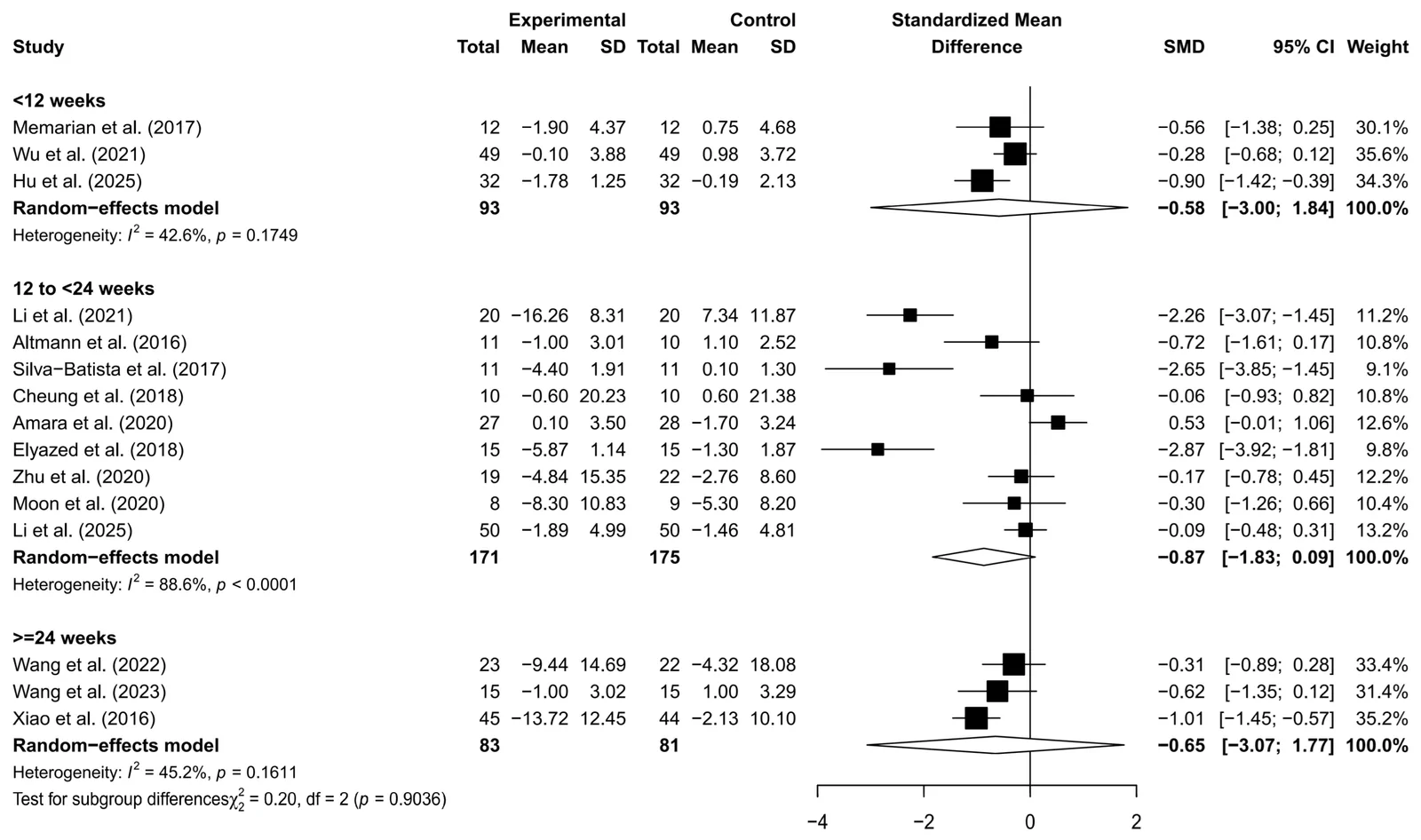
Subgroup Analysis of Intervention Duration [21,22,23,24,25,26,27,28,29,30,31,32,35,36,37].

Fifteen studies were included in the subgroup analysis by intervention duration. Three studies had intervention durations of <12 weeks [27,30,32], nine had durations of 12 to <24 weeks [21,22,24,25,26,28,31,35,36], and three had durations of ≥24 weeks [23,29,37]. For interventions lasting <12 weeks, the pooled effect estimate was SMD = −0.58 (95% CI −3.00 to 1.84; I^2^ = 42.6%). For interventions lasting 12 to <24 weeks, the pooled SMD was −0.87 (95% CI −1.83 to 0.09; I^2^ = 88.6%). For interventions lasting ≥24 weeks, the pooled effect estimate was SMD = −0.65 (95% CI −3.07 to 1.77; I^2^ = 45.2%). The test for subgroup differences was not statistically significant (χ^2^ = 0.20, df = 2, *p* = 0.9036). Detailed results are presented in Figure 7.

##### Exercise Type

**Figure 8:**
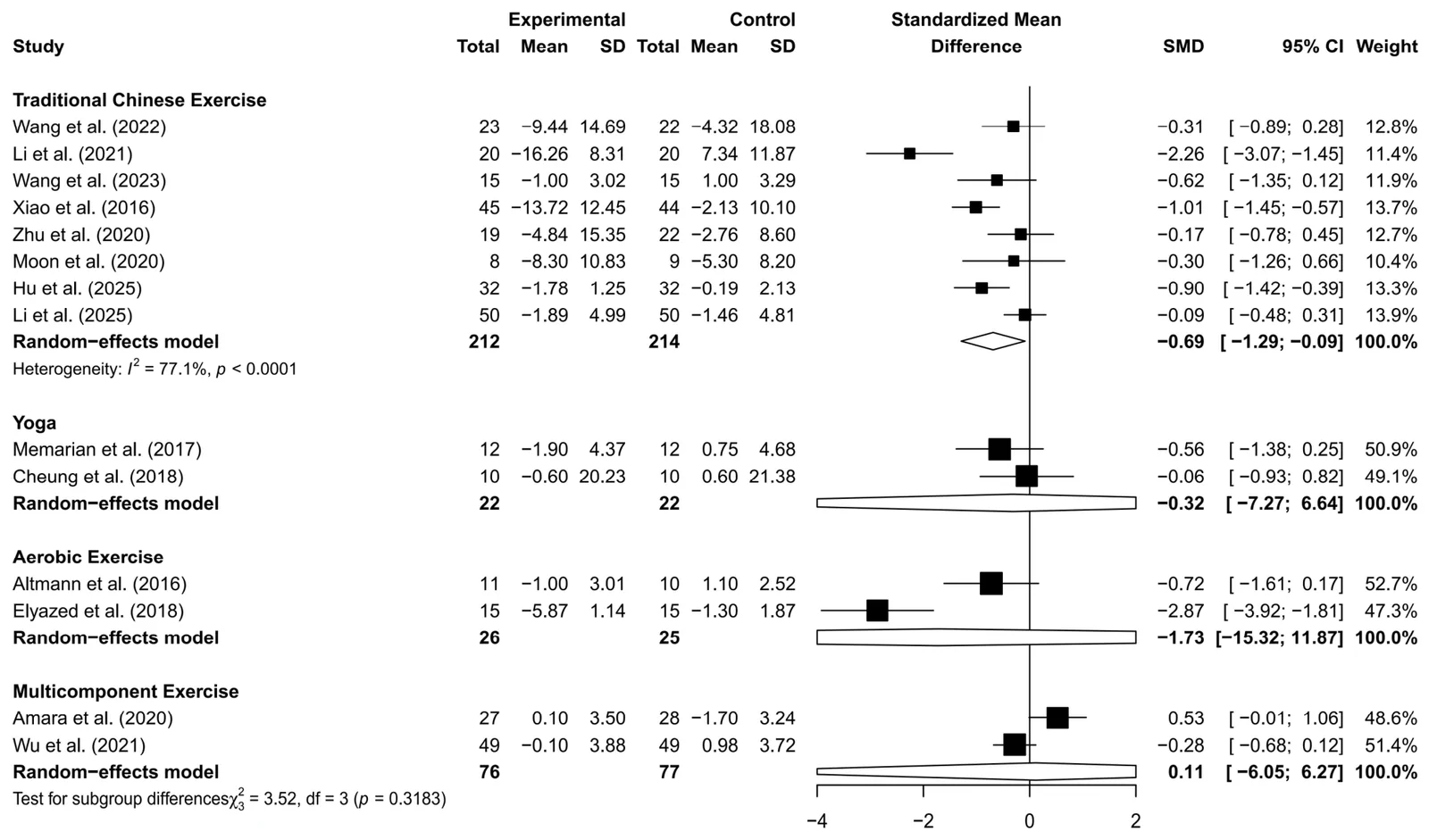
Subgroup Analysis by Type of Exercise [21,22,23,24,25,26,27,28,29,30,32,35,36,37].

Fourteen studies were included in the subgroup analysis by exercise type. Eight studies were classified as Traditional Chinese Exercise [22,23,24,27,28,29,36,37], two as Yoga [21,30], two as Aerobic Exercise [25,35], and two as Multicomponent Exercise [26,32]. One study classified as Resistance Exercise [31] represented a single-study category and was not included in the pooled subgroup analysis. For Traditional Chinese Exercise, the pooled effect estimate was SMD = −0.69 (95% CI −1.29 to −0.09; I^2^ = 77.1%). The pooled SMD was −0.32 (95% CI −7.27 to 6.64) for Yoga, −1.73 (95% CI −15.32 to 11.87) for Aerobic Exercise, and 0.11 (95% CI −6.05 to 6.27) for Multicomponent Exercise. The test for subgroup differences was not statistically significant (χ^2^ = 3.52, df = 3, *p* = 0.3183). Detailed results are presented in Figure 8.

### 3.4. Meta-Regression Results

Three separate exploratory univariable meta-regression analyses were conducted to examine the associations of session duration, weekly exercise frequency, and intervention period with the intervention effect. The session-duration and weekly-frequency models each included 13 studies [21,22,23,24,25,26,27,28,29,30,31,35,37], whereas the intervention-period model included all 15 studies [21,22,23,24,25,26,27,28,29,30,31,32,35,36,37]. Session duration was not significantly associated with the effect size (β = −0.009, SE = 0.018, 95% CI −0.049 to 0.031; *p* = 0.647; R^2^ = 0.0%; k = 13). Weekly exercise frequency was also not significantly associated with the effect size (β = 0.156, SE = 0.304, 95% CI −0.514 to 0.826; *p* = 0.619; R^2^ = 0.0%; k = 13). Similarly, no statistically significant association was observed for intervention period (β = 0.017, SE = 0.046, 95% CI −0.083 to 0.116; *p* = 0.725; R^2^ = 0.0%; k = 15). The estimated R^2^ was 0.0% for each model. Detailed results are presented in Table 2.

### 3.5. Assessment of Small-Study Effects

**Figure 9:**
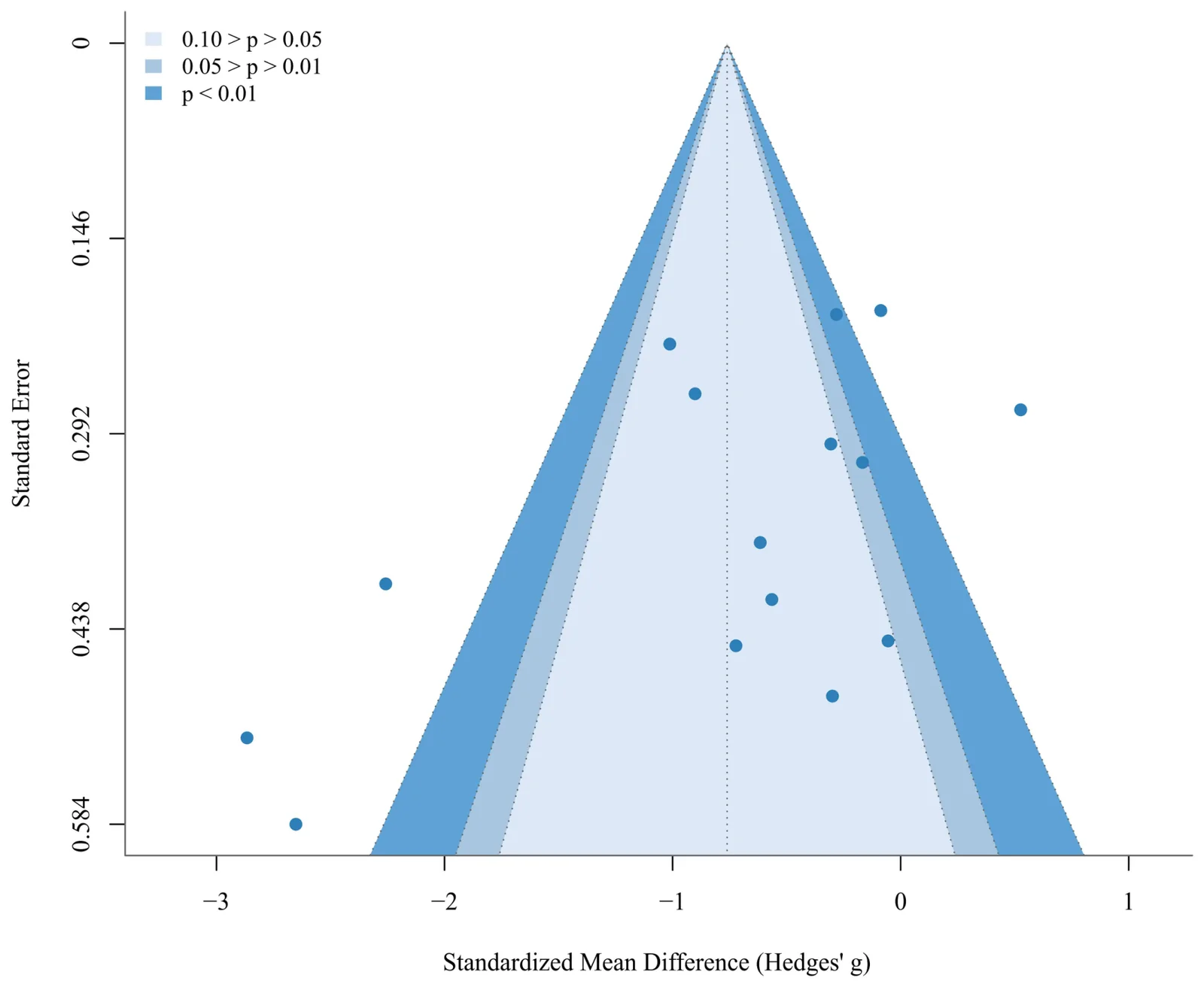
Contour-enhanced funnel plot for the primary meta-analysis.

**Figure 10:**
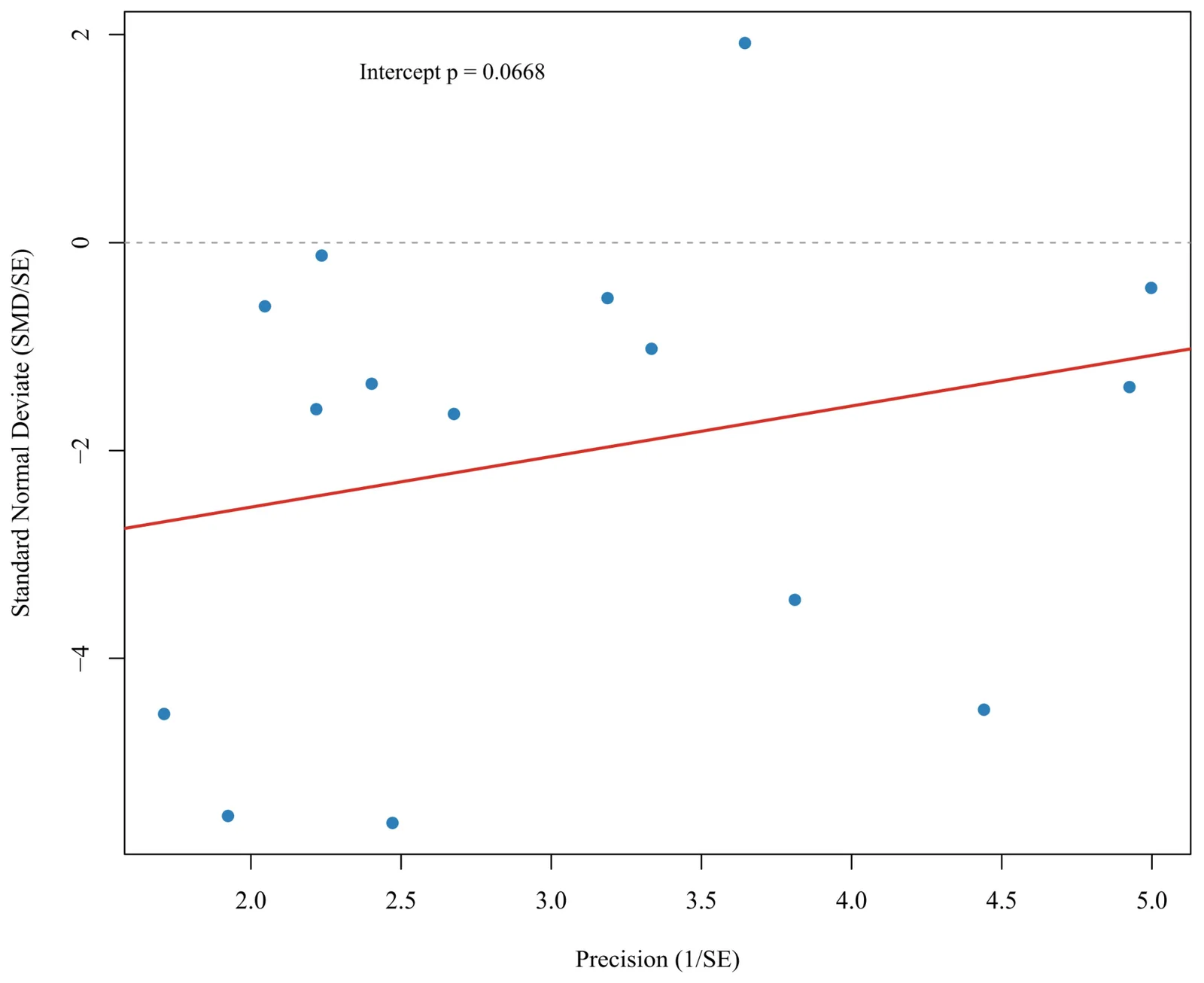
Egger regression plot for the assessment of small-study effects.

Visual inspection of the contour-enhanced funnel plot suggested some asymmetry [21,22,23,24,25,26,27,28,29,30,31,32,35,36,37]. Egger’s regression test did not reach conventional statistical significance (*p* = 0.0668). However, given the limited number of included studies and the consequently limited power of tests for funnel-plot asymmetry, small-study effects or publication bias cannot be excluded. The contour-enhanced funnel plot and Egger regression plot are presented in Figure 9 and Figure 10, respectively.

### 3.6. Certainty of Evidence

Evidence from RCTs started at high certainty and was downgraded by one level for serious risk of bias, primarily because of concerns regarding allocation concealment and blinding-related domains in studies relying on self-reported sleep outcomes. The evidence was downgraded by a further level for serious inconsistency because substantial between-study heterogeneity was observed in the primary analysis (I^2^ = 82.4%; τ^2^ = 0.6936; *p* < 0.0001) and remained substantial across instrument-stratified, sensitivity, subgroup, and meta-regression analyses. No downgrade was applied for indirectness because the included trials directly evaluated exercise-based interventions in patients with PD and assessed clinically relevant self-reported sleep outcomes. The evidence was downgraded by one level for serious imprecision because, although the 95% CI for the pooled effect did not cross the rule-of-thumb threshold for a small standardized effect (|SMD| = 0.20), the total sample size (*n* = 696) was below the approximate GRADE rule-of-thumb optimal information size of 800 participants for continuous outcomes. No downgrade was applied for publication bias because the available evidence was insufficient to establish its presence. Nevertheless, the observed funnel-plot asymmetry and the limited power of Egger’s regression test with only 15 studies mean that small-study effects or publication bias cannot be excluded. Overall, the certainty of evidence for the primary self-reported sleep outcome was rated as very low (Table 3).

## 4. Discussion

### 4.1. Summary of Evidence

The present meta-analysis indicates that exercise-based interventions are associated with improved self-reported sleep outcomes in patients with PD. This finding was supported by the PDSS/PSQI-restricted sensitivity analysis and leave-one-out analyses, which yielded consistently favorable estimates. However, substantial heterogeneity persisted across these analyses, suggesting that the variability in treatment effects was not attributable to differences in sleep measurement instruments or to any single study alone. Exploratory subgroup analyses showed no statistically significant differences according to exercise intensity, session duration, weekly frequency, intervention period, or exercise type, and univariable meta-regression identified no significant associations for session duration, weekly frequency, or intervention period. The estimated R^2^ was 0% in all three meta-regression models, providing no evidence that the examined study-level moderators explained the substantial between-study heterogeneity. Overall, the findings suggest a possible benefit of exercise-based interventions, but the influence of specific exercise characteristics remains uncertain.

The overall findings are consistent with previous evidence showing beneficial effects of exercise on sleep-related outcomes in middle-aged and older adults and in other clinical populations [38,39,40,41,42]. Nevertheless, sleep disturbances in PD arise from a complex interplay of disease-specific factors, including nocturnal motor symptoms, dopaminergic treatment, mood disturbances, autonomic dysfunction, and alterations in sleep-regulating neural systems, which may contribute to variability in treatment response. Heterogeneity may also reflect differences in disease severity, participant characteristics, exercise protocols, comparator conditions, and the sleep instruments used across trials. Although the included questionnaires assess related aspects of self-reported sleep, they capture partially distinct constructs, and their use may therefore contribute to variability in standardized effect estimates. In addition, concerns regarding allocation concealment and blinding were common among the included trials. These factors, together with substantial inconsistency and imprecision, resulted in a very low certainty of evidence according to GRADE. Thus, the findings suggest a possible benefit of exercise-based interventions for self-reported sleep outcomes in PD; however, the very low certainty of evidence precludes a specific clinical recommendation, while highlighting the need for more rigorous trials to better define the magnitude of benefit and the exercise characteristics most relevant to treatment response.

### 4.2. Potential Mechanisms Linking Exercise to Sleep Outcomes in Parkinson’s Disease

Several biological pathways may contribute to the association between exercise and improved sleep-related outcomes in Parkinson’s disease. Exercise has been proposed to enhance neuroplasticity and increase brain-derived neurotrophic factor (BDNF), while also modulating dopaminergic, serotonergic, and γ-aminobutyric acid-related signaling involved in sleep–wake regulation [43,44,45,46]. Exercise may additionally attenuate neuroinflammatory and oxidative processes implicated in both PD-related neurodegeneration and sleep disturbance. These effects could, in turn, contribute to improvements in nocturnal symptoms and sleep regulation.

Exercise may also influence mitochondrial function and endogenous antioxidant and neuroprotective pathways, which could help reduce cellular stress in dopaminergic systems [47,48,49]. Changes in BDNF, inflammatory signaling, oxidative stress, and other neuroprotective pathways have therefore been proposed as possible links between exercise and sleep-related benefits in PD [49,50,51]. Collectively, these interconnected pathways may help explain how exercise influences sleep-related outcomes in PD and highlight the multifactorial nature of the exercise–sleep relationship in this population.

### 4.3. Clinical Implications

The available evidence does not yet demonstrate a consistent dose–response pattern or a clearly preferable exercise modality for sleep-related outcomes in PD. The subgroup and meta-regression analyses were limited by the small number of available trials, small subgroup sizes, and substantial residual heterogeneity. Accordingly, the absence of statistically significant moderator effects should not be interpreted as evidence that exercise characteristics do not influence treatment response.

From a clinical perspective, the current evidence suggests a possible benefit of exercise-based interventions for self-reported sleep outcomes in PD but is insufficient to support a specific clinical recommendation for sleep-related symptoms. Where exercise is used as part of broader PD management, its prescription should be individualized according to disease severity, motor and non-motor symptoms, comorbidities, physical capacity, tolerance, accessibility, and patient preference, with appropriate attention to adherence and safety. Future trials should directly compare well-defined exercise protocols, report exercise dose and adherence consistently, and incorporate both validated patient-reported and objective sleep measures, such as actigraphy or polysomnography. Wearable and remote-monitoring technologies may further facilitate longitudinal collection of sleep, physical activity, and other health-related data beyond conventional clinical settings [52,53]. Such studies will be important for clarifying whether specific exercise characteristics are associated with differential sleep-related benefits in PD.

### 4.4. Limitations

Several limitations should be considered when interpreting the findings. First, sleep outcomes were assessed exclusively using self-reported questionnaires. Although these instruments are clinically relevant, they capture related but partially distinct aspects of sleep and may be influenced by recall, perception, and other patient-level factors. The use of different instruments also necessitated pooling effects using SMDs, which facilitates quantitative synthesis but limits direct interpretation in instrument-specific clinical units. The absence of objective sleep measures, such as actigraphy or polysomnography, further limits assessment of whether the observed improvements correspond to changes in objectively measured sleep.

Second, substantial clinical and methodological heterogeneity remained across the included trials. Differences in participant characteristics, disease severity and duration, exercise modality and dose, comparator conditions, and sleep assessment methods may all have contributed to variation in treatment effects. Although instrument-stratified, PDSS/PSQI-restricted, and leave-one-out analyses supported the overall direction of the findings, they did not materially resolve the heterogeneity. In addition, only 15 RCTs were available, and several subgroup categories contained few studies. Consequently, the subgroup analyses and study-level meta-regression had limited statistical power, and the absence of statistically significant moderator effects should not be interpreted as evidence that exercise characteristics do not influence treatment response.

Finally, the review was restricted to studies published in English or Chinese, and relevant evidence reported in other languages may therefore have been missed. A substantial proportion of the included trials were also conducted in Asian populations, particularly in mainland China and Taiwan, which may limit the generalizability of the findings to more geographically and ethnically diverse populations. Risk-of-bias concerns were common in allocation concealment and blinding-related domains, which is particularly relevant for trials relying on self-reported outcomes. Taken together, these limitations should be considered when interpreting the magnitude and generalizability of the pooled effect.

## 5. Conclusions

Exercise-based interventions may improve self-reported sleep outcomes in patients with Parkinson’s disease. However, the certainty of evidence is very low, substantial unexplained heterogeneity remains, and the available evidence is insufficient to support a specific clinical recommendation or exercise prescription for sleep-related symptoms in PD. Future adequately powered RCTs using standardized exercise protocols and both patient-reported and objective sleep measures are needed to better define the magnitude and clinical relevance of any benefit and the exercise characteristics associated with treatment response.

## Figures and Tables

**Table 1:** Characteristics of the included studies.

Author	Year	Country/Region	Sample Size (T/C)	Age, Years (T/C)	Hoehn and Yahr Stage	Exercise Intervention	Reported Intensity	Comparator	Intervention Dose	Sleep Outcome
Wang et al. [23]	2022	China	23/22	68.83 ± 4.35/67.95 ± 4.86	T/C: 1.28 ± 0.45/1.23 ± 0.40	Wu Qin Xi (mind–body exercise)	Low-to-moderate; heart rate monitored, no target range reported	Active exercise control (stretching)	90 min/session; 3 sessions/week; 24 weeks	PDSS
Li et al. [22]	2021	China	20/20	66.95 ± 7.44/66.11 ± 8.02	I–III	Mind–body exercise	Moderate as coded in the source manuscript; no numerical HR/RPE threshold reported	Usual care/no added exercise	60 min/session; 5 sessions/week; 16 weeks	PDSS
Wang et al. [29]	2023	China	15/15	72.07 ± 8.33/67.13 ± 8.33	I–III	Tai Chi (mind–body exercise)	Low-to-moderate; HR and oxygen saturation monitored, no target threshold reported	No intervention/supportive care	40 min/session; 3 sessions/week; 24 weeks	PSQI
Altmann et al. [25]	2016	USA	11/10	62.8 ± 8.6/67.8 ± 9.8	I–III	Treadmill aerobic exercise	50–75% heart-rate reserve	No-contact/no-exercise control	20–45 min/session; 3 sessions/week; 16 weeks	PSQI
Xiao et al. [37]	2016	China	45/44	68.17 ± 2.27/66.52 ± 2.13	T/C: 2.2 ± 0.21/2.1 ± 0.23	Baduanjin Qigong plus daily walking	Low intensity; mean induced HR 43–49% of predicted HRmax	Active exercise control (daily walking)	Qigong 12–15 min/session, at least daily and ≥4 sessions/week; both groups walked ≥ 30 min/day; 24 weeks	PDSS-2
Memarian et al. [30]	2017	Iran	12/12	55–75 (overall range)	I–III inclusion	Laughter yoga	Gentle/low intensity; not quantitatively reported	Usual care/standard medical treatment	45 min/session; 2 sessions/week; 8 weeks	PSQI
Silva-Batista et al. [31]	2017	Brazil	11/11	64.6 ± 9.7/64.4 ± 9.1	T/C: 2.5 ± 0.5/2.5 ± 0.4	Resistance training	Progressive RT: 10–12RM to 8–10RM to 6–8RM over 3 months	Attention control/no exercise (education and bingo)	50 min/session; 2 sessions/week; 12 weeks	PSQI
Cheung et al. [21]	2018	USA	10/10	63.5 ± 8.5/65.8 ± 6.6	T/C: 2.0 ± 0.8/2.0 ± 0.8	Hatha yoga	Gentle; no HR/RPE threshold reported	Wait-list/usual care	60 min/session; 2 sessions/week; 12 weeks	PDSS
Amara et al. [26]	2020	USA	27/28	65.33 ± 8.17/65.82 ± 5.19	II–III	Combined resistance and functional exercise	High intensity; sessions 1 and 3 targeted 10RM, session 2 used ~30% lower load	Active non-exercise control (sleep hygiene)	Session duration NR; 3 sessions/week; 16 weeks	PSQI
Elyazed et al. [35]	2018	Egypt	15/15	66.13 ± 5.66/65.27 ± 4.96	NR	Treadmill aerobic exercise	40–50% heart-rate reserve; described as moderate intensity	Active exercise control (strengthening and stretching)	40 min/session; 3 sessions/week; 12 weeks	ISI
Zhu et al. [24]	2020	China	19/22	68.53 ± 1.90/67.77 ± 1.72	II–III	Tai Chi plus routine exercise	Moderate as coded in the source manuscript; no extractable numerical threshold	Active exercise control (routine exercise)	45 min/session; 3 sessions/week; 12 weeks	PDSS
Wu et al. [32]	2021	Taiwan	49/49	63.65 ± 6.02/66.59 ± 8.61	I–II	Home-based combined aerobic + resistance exercise	Aerobic target 60–80% HRmax; resistance progressed to 65–70% RM	Usual care/no exercise	50 min/session; ≥3 sessions/week (target 150 min/week); 8 weeks	PSQI
Moon et al. [36]	2020	USA	8/9	66.4 ± 8.1/65.9 ± 5.4	Median T/C: 2 (2-2)/2 (2-2)	Six-healing-sounds Qigong	Low intensity; mild body movements, not quantified by HR/RPE	Sham/active exercise control	15–20 min home session twice daily plus one 45–60 min supervised group session/week; 12 weeks	PDSS-2
Hu et al. [27]	2025	China	32/32	67.59 ± 4.94/69.87 ± 3.42	NR	Mind–body exercise	Moderate	Active non-exercise control (conventional rehabilitation + insomnia education)	30 min/session; 5 sessions/week; 8 weeks	PSQI
Li et al. [28]	2025	China	50/50	65.43 ± 7.27/66.41 ± 7.41	T/C: 2.51 ± 0.55/2.41 ± 0.68	Mind–body exercise	Moderate	No exercise/maintained usual lifestyle	30 min/session; 5 sessions/week; 12 weeks	PSQI

Note. T, exercise intervention group; C, control group; NR, not reported; PSQI, Pittsburgh Sleep Quality Index; PDSS, Parkinson’s Disease Sleep Scale; PDSS-2, Parkinson’s Disease Sleep Scale-2; ISI, Insomnia Severity Index; HR, heart rate; HRmax, maximal heart rate; RM, repetition maximum; RPE, rating of perceived exertion; RT, resistance training.

**Table 2:** Results of Exploratory Univariable Meta-Regression Analyses.

Moderator	k	β	SE	95% CI	*p*-Value	R^2^ (%)
Session duration, min	13	−0.009	0.018	−0.049 to 0.031	0.647	0.0
Weekly frequency, sessions/week	13	0.156	0.304	−0.514 to 0.826	0.619	0.0
Intervention period, weeks	15	0.017	0.046	−0.083 to 0.116	0.725	0.0

Note. k, number of studies included in each meta-regression model; β, regression coefficient; SE, standard error; CI, confidence interval; R^2^, proportion of between-study heterogeneity explained by the moderator.

**Table 3:** Summary of Findings and GRADE Assessment for the Primary Sleep Outcome.

Outcome (Primary Outcome)	Participants (Studies)	Study Design	Effect (SMD, 95% CI)	Heterogeneity	Certainty of Evidence (GRADE)	Downgrading Decisions
Self-reported sleep outcome (sleep questionnaires; harmonized direction)	696 (15 RCTs)	RCT	−0.75 (−1.28 to −0.23)	I^2^ = 82.4% τ^2^ = 0.6936 *p* < 0.0001	VERY LOW ⊕◯◯◯	Risk of bias (−1); Inconsistency (−1); Indirectness (0); Imprecision (−1); Publication bias (0)

Note. RCT, randomized controlled trial; SMD, standardized mean difference; CI, confidence interval; I^2^, proportion of total variability attributable to between-study heterogeneity; τ^2^, estimated between-study variance. Negative SMD values favor exercise after harmonization of score directions across instruments. ⊕◯◯◯ indicates very low certainty of evidence. The certainty of evidence was downgraded for serious risk of bias, serious inconsistency, and serious imprecision. No downgrade was applied for indirectness or publication bias.

## Data Availability

The data used in this study were extracted from previously published articles, which are cited in the reference list. The extracted and analyzed datasets are available from the corresponding author upon reasonable request.

## Supplementary Materials

The following supporting information can be downloaded at https://www.mdpi.com/article/10.3390/bioengineering13091027/s1, Table S1: Complete database-specific search strategies; Figure S1: Forest plot of the instrument-stratified meta-analysis [21,22,23,24,25,26,27,28,29,30,31,32,36,37]; Figure S2: Sensitivity analysis restricted to studies using the PDSS or PSQI [21,22,23,24,25,26,27,28,29,30,31,32]; Figure S3: Leave-one-out sensitivity analysis of the primary meta-analysis [21,22,23,24,25,26,27,28,29,30,31,32,35,36,37]; Figure S4: Subgroup analysis according to comparator type [21,22,23,24,25,26,27,28,29,30,31,32,35,36,37].

## Abbreviations

The following abbreviations are used in this manuscript:

BDNF	Brain-derived neurotrophic factor
CI	Confidence interval
CNKI	China National Knowledge Infrastructure
GRADE	Grading of Recommendations Assessment, Development and Evaluation
INPLASY	International Platform of Registered Systematic Review and Meta-analysis Protocols
ISI	Insomnia Severity Index
MeSH	Medical Subject Headings
PD	Parkinson’s disease
PDSS	Parkinson’s Disease Sleep Scale
PDSS-2	Parkinson’s Disease Sleep Scale-2
PRISMA	Preferred Reporting Items for Systematic Reviews and Meta-Analyses
PSQI	Pittsburgh Sleep Quality Index
RCT	Randomized controlled trial
RoB	Cochrane Risk of Bias Tool
RPE	Rating of Perceived Exertion
SMD	Standardized mean difference
