# Supplementary material for: Effects of Exercise-Based Interventions on Self-Reported Sleep Outcomes in Parkinson’s Disease: A Systematic Review and Meta-Analysis of Randomized Controlled Trials
Source: Bioengineering (Basel). 2026 Sep 3;13(9):1027. doi: 10.3390/bioengineering13091027 (PMC13603865; doi:10.3390/bioengineering13091027)
Supplement: Supplementary file 1 [file bioengineering-13-01027-s001.zip › bioengineering-4501528-supplementary.pdf]

bioengineering

MDPI

Supplementary

Effects of Exercise-Based Interventions on Self-Reported Sleep Outcomes in Parkinson's Disease: A Systematic Review and Meta-Analysis of Randomized Controlled Trials

Chengshuo Zhang [1], Xinyuan Zhang [2], Lei Cao [2], Jiaxin Li [2], Zhengqiu Zhang [2] and Wanli Zang [2,*]

[1] School of Physical Education, Henan University of Science and Technology, Luoyang 471023, China
[2] School of Physical Education, Soochow University, Suzhou 215021, China
* Correspondence: 20244006004@stu.suda.edu.cn

Table S1. Complete database-specific search strategies.

Search period: database inception to 17 August 2026.

Database / platform	Search strategy
**PubMed**	("Parkinson Disease"[Mesh] OR parkinson*[tiab] OR "paralysis agitans"[tiab]) AND ("Exercise"[Mesh] OR "Exercise Therapy"[Mesh] OR "Exercise Movement Techniques"[Mesh] OR "Physical Conditioning, Human"[Mesh] OR "Resistance Training"[Mesh] OR "Tai Ji"[Mesh] OR "Yoga"[Mesh] OR "Qigong"[Mesh] OR exercis*[tiab] OR "physical activ*"[tiab] OR "physical training"[tiab] OR "exercise training"[tiab] OR "exercise program*"[tiab] OR "exercise programme*"[tiab] OR "exercise intervention*"[tiab] OR "exercise therap*"[tiab] OR aerobic*[tiab] OR "aerobic training"[tiab] OR "aerobic exercise*"[tiab] OR "endurance training"[tiab] OR "resistance training"[tiab] OR "resistance exercise*"[tiab] OR "strength training"[tiab] OR "strength exercise*"[tiab] OR "weight training"[tiab] OR treadmill*[tiab] OR yoga[tiab] OR "tai chi"[tiab] OR "tai ji"[tiab] OR taiji[tiab] OR taijiquan[tiab] OR "tai chi chuan"[tiab] OR qigong[tiab] OR "qi gong"[tiab] OR baduanjin[tiab] OR "ba duan jin"[tiab] OR "eight-section brocade"[tiab] OR "eight section brocade"[tiab] OR wuqinxi[tiab] OR "wu qin xi"[tiab] OR "five-animal exercise*"[tiab] OR "five animal exercise*"[tiab] OR yijinjing[tiab] OR "yi jin jing"[tiab] OR "multicomponent exercise*"[tiab] OR "multi-component exercise*"[tiab] OR "multimodal exercise*"[tiab] OR "multi-modal exercise*"[tiab]) AND (randomized controlled trial[pt] OR controlled clinical trial[pt] OR random*[tiab] OR placebo[tiab] OR randomly[tiab] OR trial[tiab]) NOT (animals[mh] NOT humans[mh])
**Web of Science Core Collection**	TS=(<br>parkinson*<br>OR "paralysis agitans"<br>)<br>AND<br>TS=(<br>exercis*<br>OR "physical activ*"<br>OR "physical training"<br>OR "exercise training"<br>OR "exercise program*"<br>OR "exercise programme*"<br>OR "exercise intervention*"<br>OR "exercise therap*"<br>OR aerobic*<br>OR "aerobic training"<br>OR "aerobic exercise*"<br>OR "endurance training"<br>OR "resistance training"<br>OR "resistance exercise*"<br>OR "strength training"

Database / platform	Search strategy
	OR "strength exercise*"<br>OR "weight training"<br>OR treadmill*<br>OR yoga<br>OR "tai chi"<br>OR "tai ji"<br>OR taiji<br>OR taijiquan<br>OR "tai chi chuan"<br>OR qigong<br>OR "qi gong"<br>OR baduanjin<br>OR "ba duan jin"<br>OR "eight-section brocade"<br>OR "eight section brocade"<br>OR wuqinxi<br>OR "wu qin xi"<br>OR "five-animal exercise*"<br>OR "five animal exercise*"<br>OR yijinjing<br>OR "yi jin jing"<br>OR "multicomponent exercise*"<br>OR "multi-component exercise*"<br>OR "multimodal exercise*"<br>OR "multi-modal exercise*"<br>)<br>AND<br>(<br>TI=(<br>random*<br>OR "randomized controlled trial*"<br>OR "randomised controlled trial*"<br>OR "controlled clinical trial*"<br>OR "clinical trial*"<br>OR placebo*<br>)<br>OR<br>AB=(<br>random*<br>OR "randomized controlled trial*"<br>OR "randomised controlled trial*"<br>OR "controlled clinical trial*"<br>OR "clinical trial*"<br>OR placebo*<br>)<br>)
**Scopus**	TITLE-ABS-KEY (parkinson* OR "paralysis agitans") AND TITLE-ABS-KEY (exercis* OR "physical activity" OR "physical training" OR "exercise training" OR "exercise program" OR "exercise programme" OR "exercise intervention" OR "exercise therapy" OR aerobic* OR "endurance training" OR "resistance training" OR "resistance exercise" OR "strength training" OR "strength exercise" OR "weight training" OR treadmill* OR yoga OR "tai chi" OR "tai ji" OR taiji OR taijiquan OR "tai chi chuan" OR qigong OR "qi gong" OR baduanjin OR "ba duan jin" OR "eight section brocade" OR wuqinxi OR "wu qin xi" OR "five animal exercise" OR yijinjing OR "yi jin jing" OR "multicomponent exercise" OR "multi-component exercise" OR "multimodal exercise" OR "multi-modal exercise") AND TITLE-ABS-KEY (random* OR "randomized controlled trial" OR "randomised controlled trial" OR "controlled clinical trial" OR "controlled trial" OR "clinical trial" OR placebo*)
**CENTRAL (Cochrane Library)**	**Search line Search expression Hits**<br>#1 MeSH descriptor: [Parkinson Disease] explode all trees 6736

Database / platform	Search strategy
	#2 (parkinson* OR "paralysis agitans"):ti,ab,kw 16473<br>#3 #1 OR #2 16473<br>#4 MeSH descriptor: [Exercise] explode all trees 44224<br>#5 MeSH descriptor: [Exercise Therapy] explode all trees 25609<br>#6 MeSH descriptor: [Exercise Movement Techniques] explode all trees 4266<br>#7 MeSH descriptor: [Resistance Training] explode all trees 6864<br>#8 MeSH descriptor: [Tai Ji] explode all trees 685<br>#9 MeSH descriptor: [Yoga] explode all trees 1416<br>#10 MeSH descriptor: [Qigong] explode all trees 206<br>#11 (<br>exercis*<br>OR "physical activity"<br>OR "physical activities"<br>OR "physical training"<br>OR "exercise training"<br>OR "exercise program"<br>OR "exercise programs"<br>OR "exercise programme"<br>OR "exercise programmes"<br>OR "exercise intervention"<br>OR "exercise interventions"<br>OR "exercise therapy"<br>OR aerobic*<br>OR "endurance training"<br>OR "resistance training"<br>OR "resistance exercise"<br>OR "resistance exercises"<br>OR "strength training"<br>OR "strength exercise"<br>OR "strength exercises"<br>OR "weight training"<br>OR treadmill*<br>OR walk*<br>OR cycl*<br>OR dance*<br>OR "balance training"<br>OR "balance exercise"<br>OR "balance exercises"<br>OR stretch*<br>OR yoga<br>OR "tai chi"<br>OR "tai ji"<br>OR taiji<br>OR taijiquan<br>OR "tai chi chuan"<br>OR qigong<br>OR "qi gong"<br>OR "health qigong"<br>OR baduanjin<br>OR "ba duan jin"<br>OR "eight section brocade"<br>OR wuqinxi<br>OR "wu qin xi"<br>OR "five animal exercise"<br>OR "five animal exercises"<br>OR yijinjing<br>OR "yi jin jing"<br>OR "traditional chinese exercise"<br>OR "traditional chinese exercises"

Database / platform	Search strategy
	OR "multicomponent exercise"<br>OR "multicomponent exercises"<br>OR "multimodal exercise"<br>OR "multimodal exercises"<br>):ti,ab,kw 353355<br>#12 #4 OR #5 OR #6 OR #7 OR #8 OR #9 OR #10 OR #11 354316<br>#13 #3 AND #12 3979<br>#14 #13 in Trials 3907
**CNKI**	TKA=('帕金森病'+'帕金森氏病'+'震颤麻痹'+'Parkinson disease'+'Parkinsons disease')<br>AND<br>TKA=('运动训练'+'运动干预'+'运动疗法'+'运动治疗'+'体育锻炼'+'有氧运动'+'有氧训练'+'抗阻运动'+'抗阻训练'+'阻力训练'+'力量训练'+'跑台训练'+'太极'+'太极拳'+'气功'+'健身气功'+'八段锦'+'五禽戏'+'易筋经'+'瑜伽'+'多组分运动'+'多模式运动'+'exercise training'+'exercise intervention'+'exercise therapy'+'aerobic exercise'+'resistance training'+'strength training'+'treadmill'+'Tai Chi'+'Qigong'+'Baduanjin'+'Yoga')<br>AND<br>TKA=('随机对照试验'+'随机对照'+'随机分组'+'随机分配'+'随机临床试验'+'RCT'+'randomized controlled trial'+'randomised controlled trial')

Note. Search syntax was adapted to the indexing and field structure of each database. CENTRAL was restricted to the Trials database at the final search step. Search counts are shown only where they were supplied in the original search record.

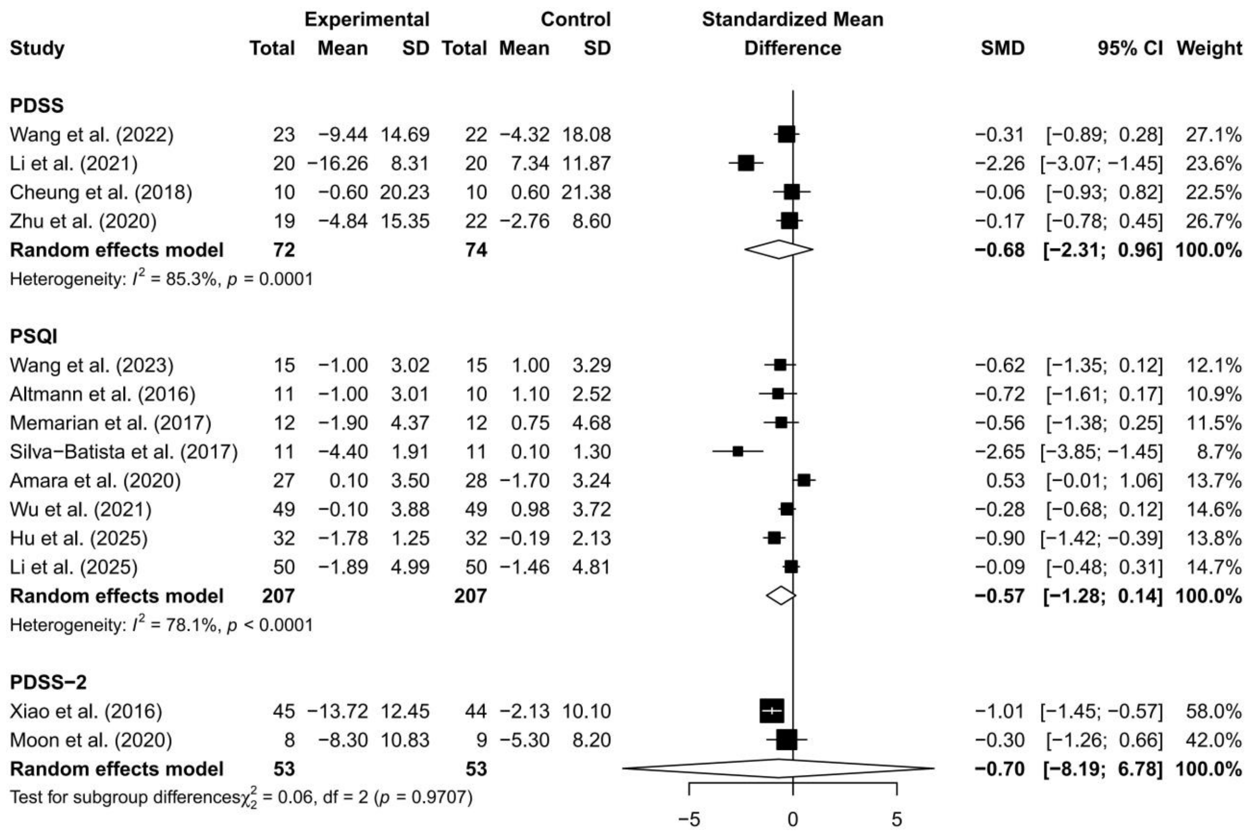

Figure S1. Forest plot of the instrument-stratified meta-analysis [21–32,36,37].

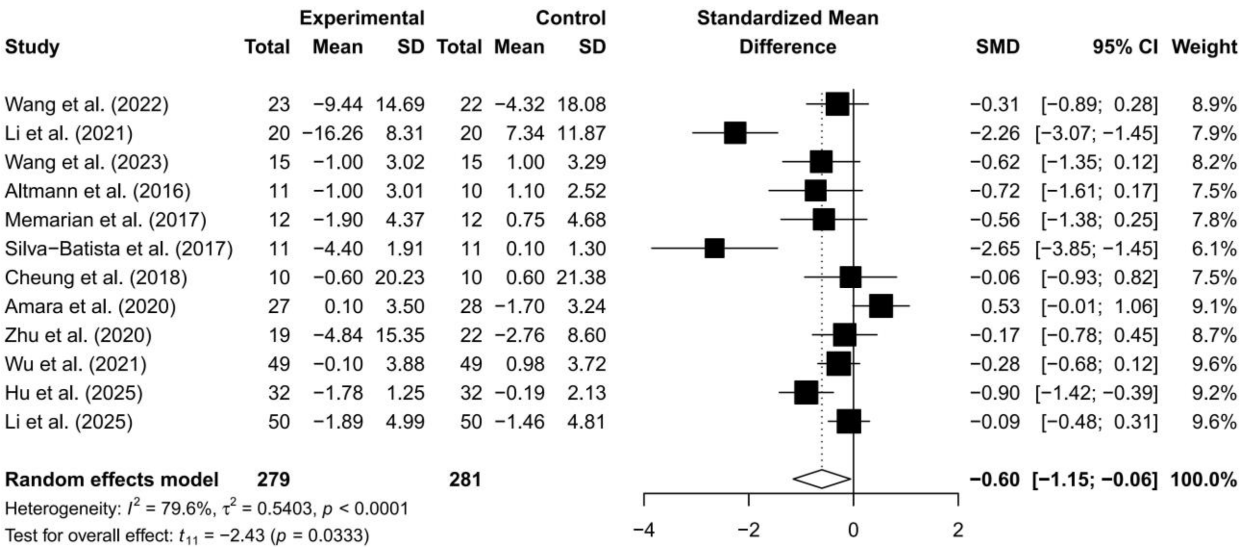

Figure S2. Sensitivity analysis restricted to studies using the PDSS or PSQI [21–32].

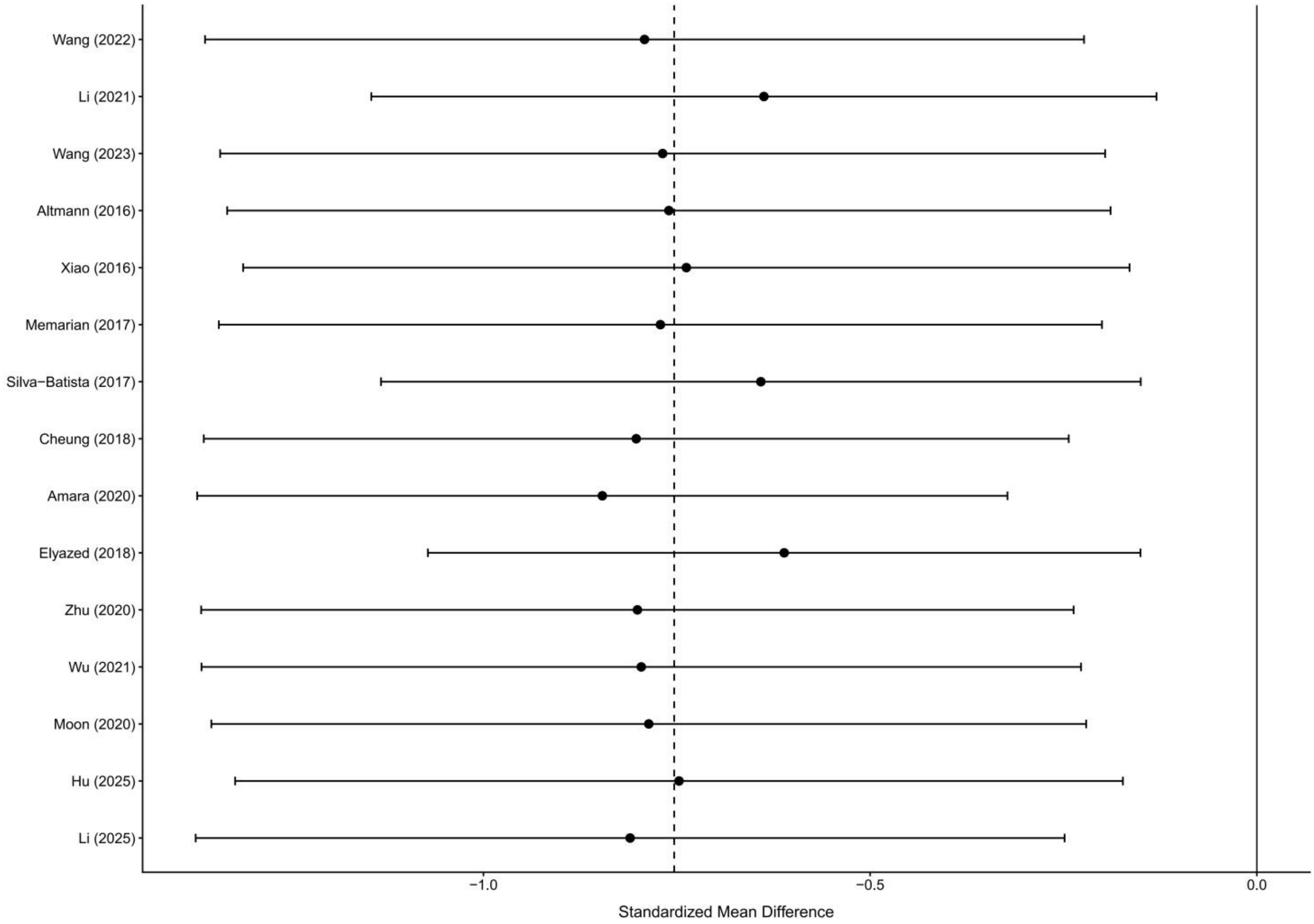

Figure S3. Leave-one-out sensitivity analysis of the primary meta-analysis [21–32,35–37].

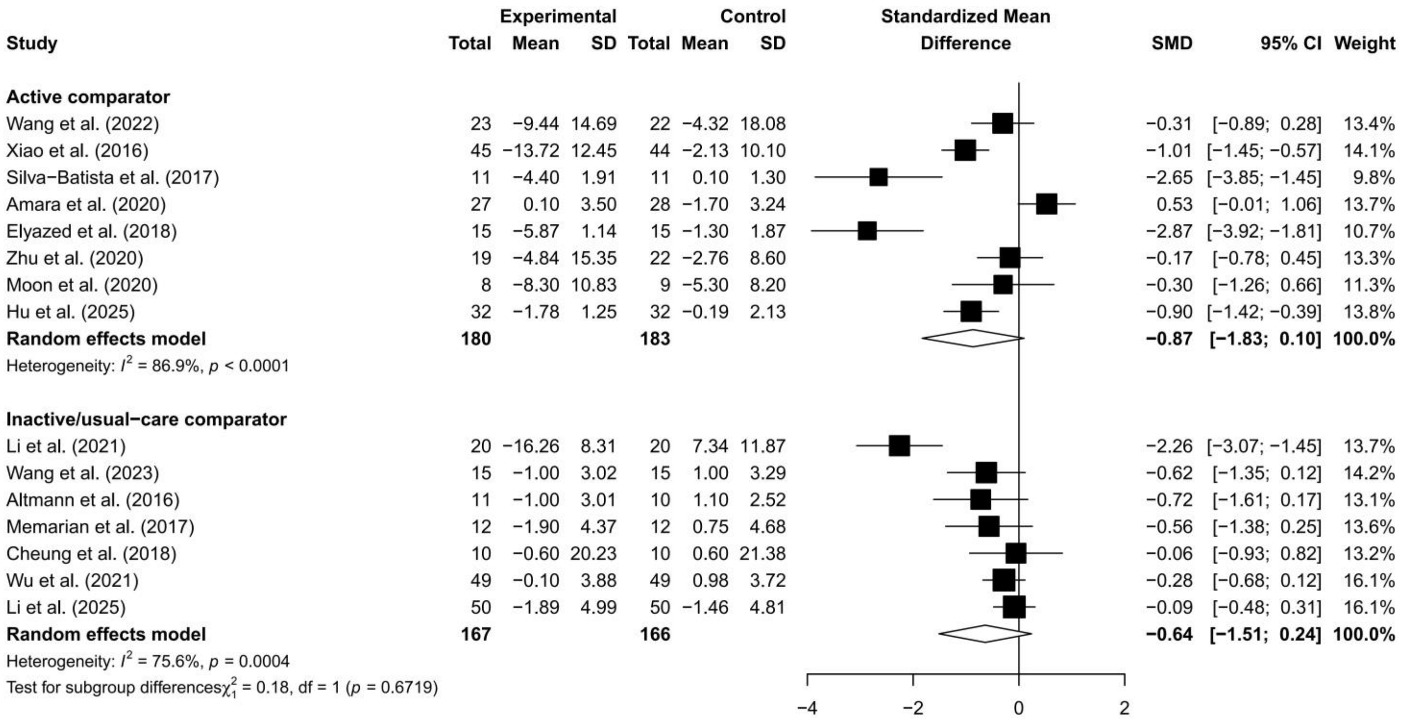

Figure S4. Subgroup analysis according to comparator type [21–32,35–37].
